# FAMA-DET: A Frequency-Domain Adaptive Multi-Scale Attention Detection Network for Aircraft Target Detection in Optical Remote Sensing Images

**DOI:** 10.3390/s26103236

**Published:** 2026-05-20

**Authors:** Lan Ma, Mingyang Peng, Yun Luo, Yujie Pi

**Affiliations:** College of Foreign Languages, National University of Defense Technology, Nanjing 210039, China; malan17@nudt.edu.cn (L.M.); pengmingyang21@nudt.edu.cn (M.P.); piyujiepyj._nudt@nudt.edu.cn (Y.P.)

**Keywords:** aircraft target detection, remote sensing image, multi-scale feature fusion, attention mechanism, frequency-domain convolution, YOLO

## Abstract

Aircraft target detection in optical remote sensing imagery is hindered by severe scale variation, cluttered backgrounds, and the limited capacity of the spatial-domain convolution to represent frequency-selective target features. We propose FAMA-DET, a frequency-domain adaptive detection framework built on YOLO11, which pursues a unified design principle of content-adaptive spectral representation across all architectural levels. The Frequency-Domain Adaptive Cross-Stage Feature Extractor (FDACFE) replaces static kernels with frequency-domain parameterised convolution driven by learnable DFT basis vectors, enabling differentiated perception of high-frequency edge details and low-frequency semantic components. The Soft-Aligned Bidirectional Feature Pyramid Network (SABFPN) eliminates upsampling amplitude distortion through scale-normalised interpolation and enriches cross-scale fusion with multi-receptive-field textural modelling. The Adaptive Multi-Scale Recalibrated Decoupled Detection Head (AMRDDHead) embeds multi-scale channel recalibration into both localisation and classification branches to suppress background redundancy and reinforce discriminative activations. On MAR20, FAMA-DET improves mAP50 and mAP50-95 over the YOLO11n baseline by 1.8% and 1.6% at only 5.4 GFLOPs, while maintaining real-time throughput of 109.7 FPS. Under zero-shot cross-domain transfer to CORS-ADD, FAMA-DET achieves the highest mAP50 of 93.3% among all compared methods, surpassing RT-DETR-R18 in mAP50 while using 91.0% fewer GFLOPs, confirming that frequency-domain adaptive design yields both strong generalisation and deployment efficiency.

## 1. Introduction

Aircraft target detection constitutes a fundamental research topic in intelligent interpretation of remote sensing imagery, with direct applications in military reconnaissance, civil aviation monitoring, and battlefield situational awareness [1]. The continuing advancement of sub-metre-resolution earth observation satellites has made large-scale acquisition of high-resolution imagery practically feasible; however, precisely localising and classifying aircraft of diverse types against complex backgrounds and under heterogeneous sensor conditions remains a core bottleneck constraining the practical deployment of intelligent perception systems.

Prior to the rise of deep learning, aircraft detection relied predominantly on handcrafted features and conventional image processing pipelines [2,3]. These early approaches achieved reasonable localisation in controlled scenarios, but could not simultaneously satisfy the requirements of content-adaptive feature representation, reliable multi-scale fusion, and discriminative capacity under high inter-class visual similarity.

Deep learning has since driven rapid progress in this domain. YOLO-series detectors have become the dominant framework owing to their favourable accuracy and efficiency trade-off, with subsequent work addressing attention-augmented feature extraction, lightweight deployment, and fine-grained recognition under pose and background variation [4,5,6,7,8]. Transformer-based detectors and multi-modal fusion approaches further extend detection capability at a higher computational cost [9,10]. Representative benchmarks underpinning these advances include the MAR20 military aircraft recognition dataset [1] and CORS-ADD [11], which together provide a solid empirical foundation for developing robust and generalisable detection algorithms.

Despite this notable progress, several persistent limitations remain unresolved in existing methods. Conventional spatial-domain convolution applies a fixed frequency response uniformly to all inputs, providing limited discriminative capacity against cluttered backgrounds and low-contrast targets. Standard feature pyramid networks further introduce scale-induced amplitude distortion through nearest-neighbour upsampling during cross-scale fusion, degrading small-target localisation accuracy. Moreover, conventional detection heads lack adaptive channel recalibration for the distinct perceptual demands of regression and classification, leading to degraded performance under high inter-class visual similarity. These limitations span all architectural levels and collectively constrain both in-domain accuracy and cross-domain generalisation.

To address these challenges, we propose the Frequency-Domain Adaptive Multi-Scale Attention Detection Network (FAMA-DET), built on a unified design principle of content-adaptive spectral representation that is embedded coherently across the backbone, neck, and detection head. The main contributions are summarised as follows:

- We propose a Frequency-Domain Adaptive Cross-Stage Feature Extractor (FDACFE), which replaces static convolutional kernels with frequency-domain parameterised convolution driven by learnable DFT basis vectors and content-adaptive modulation, enabling input-dependent frequency-selective feature extraction while simultaneously reducing computational overhead relative to the baseline.
- We design a Soft-Aligned Bidirectional Feature Pyramid Network (SABFPN), which eliminates scale-induced amplitude distortion in the top-down path via inverse-square-of-scale normalisation, and enriches multi-receptive-field textural modelling in the bottom-up path, improving cross-scale feature alignment without increasing GFLOPs.
- We construct an Adaptive Multi-Scale Recalibrated Decoupled Detection Head (AMRDDHead) that embeds multi-scale channel recalibration independently into both the regression and classification branches, aggregating multi-receptive-field statistics to suppress background redundancy and enhance fine-grained discrimination for visually similar aircraft categories.
- We conduct extensive experiments on the MAR20 in-domain benchmark and zero-shot cross-domain transfer to CORS-ADD. FAMA-DET improves mAP50 and mAP50-95 over the YOLO11n baseline by 1.8% and 1.6% at only 5.4 GFLOPs while maintaining real-time throughput of 109.7 FPS, and achieves the highest mAP50 of 93.3% among all compared methods under cross-domain transfer, surpassing RT-DETR-R18 in mAP50 while using 91.0% fewer GFLOPs.

## 2. Related Work

### 2.1. Aircraft Detection in Optical Remote Sensing Imagery

Early work relied on handcrafted features: Zhang et al. [2] combined local contrast with global sparse representation for saliency-based localisation, and Zhang et al. [3] reduced annotation dependency via weakly supervised coupled CNNs, though both remained constrained by fixed feature representations.

Deep learning shifted the field toward single-stage detectors. RSI-YOLO [4] and CNTR-YOLO [5] embed channel-spatial or ConvNeXt-Transformer attention for multi-scale discrimination; YOLO-Extract [12] combines coordinate attention with mixed dilated convolution for dense small-target scenarios. Lightweight variants [6,13] address resource-constrained deployment. Fine-grained recognition has been pursued via triple-branch feature selection and exponential moving average slide loss in FGA-YOLO [7], dense-window self-attention and Gaussian-cosine angle-aware loss in SPOD-YOLO [8], and context-aware background modelling in YOLM [14]. Beyond YOLO, Zhuang et al. [15] integrate GAN-generated features into EfficientDet, and Şengül and Adem [16] benchmark YOLOv7, YOLOv8, and RT-DETR across 43 categories, finding that inter-class confusion and class imbalance persist across architectures. RT-DETR [10] improves fine-grained recall at substantially higher computational cost, and optical–SAR fusion extends detection to multi-modal scenarios [9].

### 2.2. Feature Pyramid Networks for Multi-Scale Fusion

FPN [17] establishes the standard top-down semantic propagation path with lateral connections. PANet [18] adds a bottom-up path to shorten the distance between low- and high-level features, forming the basis of YOLO11’s neck, and BiFPN [19] introduces learnable weighted fusion via repeated stacking. On the semantic alignment side, LR-FPN [20] addresses low-level localisation loss through shallow position extraction and context interaction modules; BAFPN [21] aligns spatial position and shape in the bottom-up path while suppressing top-level aliasing; GPANet [22] applies gated path selection with soft-switching dilated convolution to expand receptive fields; and Ke et al. [23] combine Swin Transformer with a feature-enhancement FPN for semantic recalibration under SAR speckle-noise backgrounds. None of these methods address the amplitude distortion introduced by the upsampling operator itself, which is the bottleneck targeted by SABFPN.

### 2.3. Frequency-Domain Convolution Methods

FFC [24] partitions channels into a local spatial branch and a global branch using real-valued FFT to achieve image-wide receptive fields within a single layer. GFNet [25] replaces self-attention with learnable 2D FFT-domain filters, matching attention-based token mixing at lower cost. Both apply frequency operations via fixed or globally shared bases, without adapting spectral emphasis to individual input content.

### 2.4. YOLO11 Baseline

YOLO11 [26] adopts C3k2 as its backbone module, PAFPN with C2PSA as its neck, and a decoupled task-aligned detection head, surpassing YOLOv9 and YOLOv10 in parameter efficiency. Three limitations constrain its performance on optical remote sensing aircraft detection: the fixed-kernel spectral response of C3k2 cannot adapt to input-dependent frequency content; nearest-neighbour upsampling in PAFPN introduces amplitude distortion during cross-scale fusion; and the detection head lacks branch-specific channel recalibration under high inter-class visual similarity. Zhang and Wang [27] and Chen et al. [28] introduce attention and channel recalibration modules into YOLO11 and YOLOv9, respectively, but both remain within the spatial-domain paradigm.

## 3. Method

**Figure 1:**
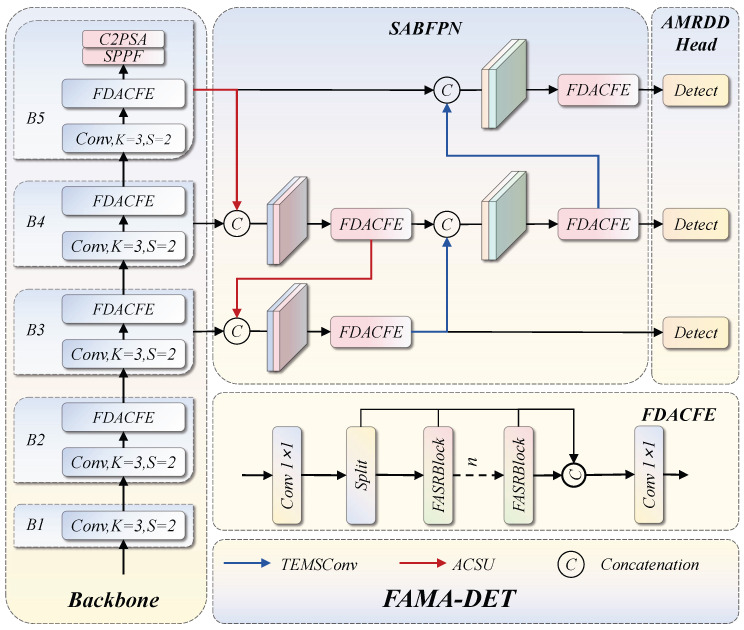
Overall architecture of the FAMA-DET algorithm.

Aircraft targets in optical remote sensing imagery exhibit simultaneous variation in scale, orientation, and spectral signature. A content-adaptive spectral response introduced at the backbone level is only effective if the subsequent fusion stage preserves rather than distorts it, and the preserved multi-scale representations are only fully exploited if the detection head can selectively recalibrate channels for the distinct perceptual demands of regression and classification. FAMA-DET is designed around this dependency through three coordinated modules, whose interconnection is depicted in Figure 1. In the backbone, FDACFE produces multi-scale feature maps $\left\{F_{3},F_{4},F_{5}\right\}$ with content-adaptive spectral responses; SABFPN then fuses these across scales via amplitude-normalised upsampling and multi-receptive-field downsampling to yield $\left\{N_{3}^{\prime },N_{4}^{\prime },N_{5}^{\prime }\right\}$; finally, AMRDDHead applies branch-specific channel recalibration before decoupled regression and classification.

### 3.1. Frequency-Domain Adaptive Cross-Stage Feature Extractor

**Figure 2:**
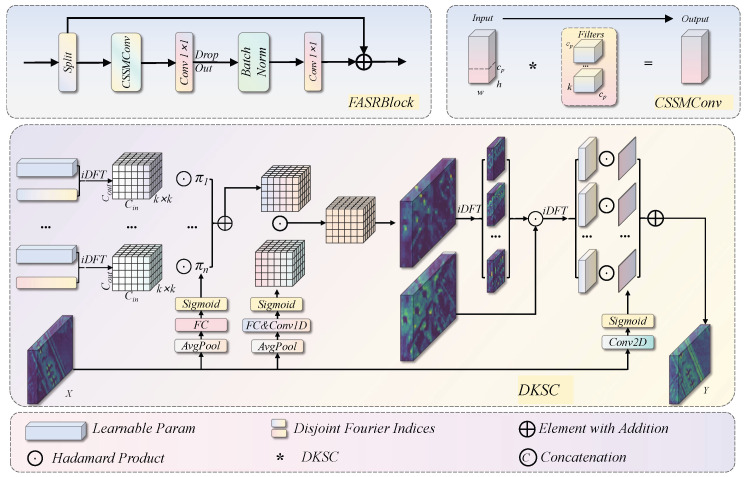
Structure of the FDACFE module. The diagram illustrates the overall architecture of FDACFE with FASRBlock as the fundamental computational unit, together with CSSMConv and DKSC, and the feature transmission pathways.

The standard C3k2 module relies on fixed static convolutional kernels whose weights remain invariant to all input feature maps after training, restricting the model’s ability to adapt to input content. From a signal processing perspective, a fixed kernel corresponds to a single invariant frequency response applied uniformly to every input. It is therefore inherently unable to selectively amplify task-relevant spectral components, such as high-frequency boundary details of aircraft outlines, or to attenuate low-frequency background clutter in a content-dependent manner. FDACFE addresses this limitation by integrating frequency-domain adaptive dynamic convolution with a partial channel acceleration strategy, as illustrated in Figure 2.

Building upon the cross-stage feature aggregation concept of C3k2, FDACFE replaces the standard Bottleneck with FASRBlock. For an input feature map *X*, a 1×1 convolution $\phi_{1}\left(\cdot \right)$ first expands the channel dimension to 2c, where $c=\lfloor C_{2}\cdot e\rfloor$ and *e* is the expansion ratio. The expanded feature map is then split along the channel dimension into two branches—$y_{0}$ and $y_{1}$. Each branch is passed through *n* serially stacked FASRBlock units for spatial-frequency-aware feature transformation. All intermediate outputs are concatenated along the channel dimension and fused by a 1×1 convolution $\phi_{2}\left(\cdot \right)$:

(1) $$Y=\phi_{2}\;\left(\mathrm{Cat}\;\left[y_{0},\;y_{1},\;F_{1}\left(y_{1}\right),\;F_{2}\;\left(F_{1}\left(y_{1}\right)\right),\;\ldots ,\;F_{n}\left(\cdots \right)\right]\right)$$

Each FASRBlock performs three operations in sequence: frequency-aware spatial mixing via CSSMConv, a channel-wise MLP transformation (consisting of a 1×1 convolution for expansion followed by another for reduction), and a residual addition. The integrated residual update takes the following form:

(2) $$X^{l+1}=X^{l}+\mathrm{DropPath}\;\left(\mathrm{MLP}\;\left(F_{\mathrm{CSSMC}}\;\left(X^{l}\right)\right)\right)$$

where $F_{\mathrm{CSSMC}}\left(\cdot \right)$ denotes the CSSMConv operation and DropPath(·) is stochastic depth regularisation to prevent overfitting in deep networks.

CSSMConv adopts a partial parameterisation strategy to reduce computational overhead. For an input feature map with *C* channels, only the first $c^{\prime }=\left\lfloor C/r_{\mathrm{div}}\right\rfloor$ channels are passed through the complete DKSC pipeline, while the remaining $C-c^{\prime }$ channels are transmitted unchanged. The two parts are then concatenated to restore the original channel dimension:

(3) $$F_{\mathrm{CSSMC}}\left(X\right)=\mathrm{Cat}\;\left(\mathrm{DKSC}\left(X_{:c^{\prime }}\right),\;X_{c^{\prime }:}\right)$$

This selective processing confines the frequency-domain transformation to the channels most responsive to spectral variation, incurring an extra computational cost of only $1/r_{\mathrm{div}}$ relative to full-channel processing.

DKSC parameterises convolutional kernels in the frequency domain using learnable DFT basis vectors. A content-adaptive Kernel Selection Mechanism (KSM) then aggregates these basis vectors into input-dependent convolution weights. The procedure consists of four steps.

The convolutional kernel parameterisation is defined natively in the real-valued frequency domain. Concretely, to determine the structure of the frequency-domain basis decomposition, a reference spatial kernel $W\in R^{C_{\mathrm{out}}\times C_{\mathrm{in}}\times k\times k}$ is reshaped to $R^{C_{\mathrm{out}}k\times C_{\mathrm{in}}k}$ and mapped to the frequency domain via 2-D real-valued FFT to obtain $\hat{W}$, which defines the dimensionality and layout of the frequency-domain parameter space.

The actual trainable parameters are *n* groups of DFT basis weights ${\left\{d_{i}\right\}}_{i=1}^{n}$, which are initialised and optimised directly in this real-valued frequency domain. They are not spatial-domain parameters that are subsequently transformed; gradient updates act on $\left\{d_{i}\right\}$ in Fourier space throughout training.

Kernel attention coefficients $\alpha \in R^{C\times n}$ are generated by a lightweight network applied to global average-pooled features, enabling input-adaptive frequency-domain weight aggregation:

(4) $${\hat{W}}^{\mathrm{agg}}=\sum_{i=1}^{n}\alpha_{i}\cdot d_{i}$$

The aggregated frequency-domain weights are mapped back to the spatial domain via inverse FFT to obtain $W_{\mathrm{agg}}=F_{2D}^{-1}\left({\hat{W}}^{\mathrm{agg}}\right)$. This operation is an analytic closed-form mapping that is numerically stable and invertible by construction given bounded basis vectors.

The resulting spatial-domain weights are then jointly modulated by three attention maps produced by the global KSM: spatial attention $A_{s}$, channel attention $A_{c}$, and filter attention $A_{f}$. In addition, a fine-grained spatial modulation coefficient $A_{\mathrm{local}}$ is generated by the local KSM. The final dynamic convolution kernel is:

(5) $$W^{*}=A_{s}⊙A_{c}⊙A_{f}⊙W_{\mathrm{agg}}⊙A_{\mathrm{local}}$$

where ⊙ denotes the Hadamard product along the corresponding dimensions.

Before entering DKSC, the input features are first passed through a Frequency Band Modulation module (FBM), which decomposes them into multiple frequency sub-bands. The sub-band boundary positions are fixed to partition the spectrum into low-, mid-, and high-frequency segments, while the per-band reweighting coefficients remain fully learnable. This further enhances the network’s selective perception of specific frequency components. The final convolution output is then computed as:

(6) $$Z=W_{\mathrm{reshape}}^{*}{*}_{\mathrm{group}}\mathrm{FBM}\left(X\right)+b$$

where ${*}_{\mathrm{group}}$ denotes grouped depthwise separable convolution and $W_{\mathrm{reshape}}^{*}$ is the aggregated weight reshaped to the format compatible with grouped convolution.

### 3.2. Soft-Aligned Bidirectional Feature Pyramid Network

**Figure 3:**
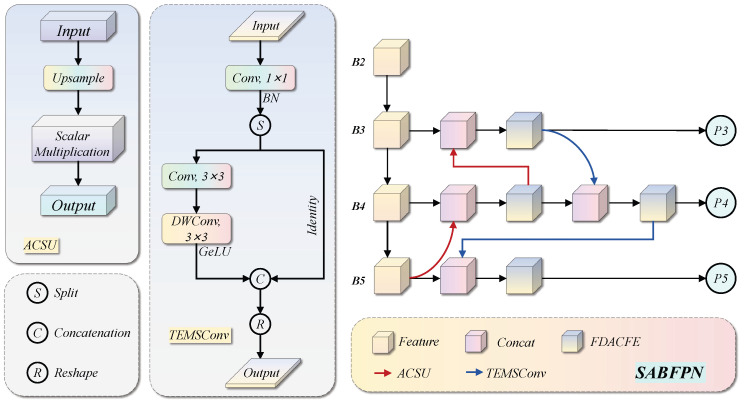
Structure of the SABFPN module. The diagram illustrates the network topology and feature interaction pattern of SABFPN, showing how ACSU and TEMSConv are incorporated into the top-down and bottom-up bidirectional fusion paths, respectively.

Cross-scale feature fusion in conventional PAFPN suffers from two geometric misalignment problems. In the top-down path, nearest-neighbour interpolation duplicates pixel values without accounting for the scale-induced amplitude difference between adjacent feature levels. As a result, high-level semantic features dominate low-level positional features during concatenation. In the bottom-up path, standard strided convolution captures only a single fixed receptive field at each stride step, which is insufficient for representing the morphologically diverse aircraft categories in MAR20 that span widely varying aspect ratios and scales. To remedy these issues, SABFPN replaces the conventional upsampling operation with ACSU and reconstructs the downsampling path with TEMSConv, as depicted in Figure 3. ACSU directly targets the amplitude distortion problem via scale-normalised compensation, while TEMSConv enriches the downsampling path with multi-receptive-field textural modelling to capture the diverse morphological patterns of aircraft categories.

The core improvement of SABFPN lies in the systematic replacement of upsampling and downsampling operators in both FPN fusion paths. In the top-down path, a high-level feature map $P_{i+1}$ is soft-upsampled by ACSU before being concatenated with the backbone feature $F_{i}$ at the corresponding scale; in the bottom-up path, low-level features undergo strided downsampling via TEMSConv before being concatenated with the fused features of the upper level. The complete bidirectional feature aggregation process is as follows:

(7) $$N_{i}=H\;\left(\mathrm{Cat}\;\left(U_{↑}\left(N_{i+1}\right),\;F_{i}\right)\right),\;i\in \left\{3,4\right\}$$

(8) $$N_{i}^{\prime }=H\;\left(\mathrm{Cat}\;\left(U_{↓}\left(N_{i-1}^{\prime }\right),\;N_{i}\right)\right),\;i\in \left\{4,5\right\}$$

where $U_{↑}\left(\cdot \right)$ denotes the ACSU soft-interpolation upsampling operator, $U_{↓}\left(\cdot \right)$ denotes the TEMSConv strided downsampling operator, H(·) is the cross-stage feature aggregation module (C3k2), and $N_{i}$ and $N_{i}^{\prime }$ are the output feature maps of the top-down and bottom-up paths at scale *i*, respectively.

Nearest-neighbour upsampling replicates each source pixel across an s×s block, inflating the aggregate feature energy by a factor of $s^{2}$. ACSU corrects this by dividing the upsampled output by $s^{2}$, restoring the per-pixel expectation to match that of the original feature map. This prevents high-level semantic features from artificially dominating low-level positional features due to scale-induced amplitude inflation rather than semantic relevance. The operation is formalised as:

(9) $$U_{↑}\left(X\right)=\frac{1}{s^{2}}\cdot \mathrm{NearestUpsample}\left(X,\;s\right)$$

where *s* is the upsampling factor.

TEMSConv enhances the downsampling path on the basis of the classic GSConv. The input feature map is first projected to a C/2-dimensional subspace via a standard convolution $\phi_{s}\left(\cdot \right)$, then two serially stacked depthwise separable convolutions generate differential textural feature branches. The two branches are concatenated and thoroughly mixed by a channel shuffle operation to fuse features from different receptive fields:

(10) $$U_{↓}\left(X\right)=\mathrm{Shuffle}\;\left(\mathrm{Cat}\;\left(\phi_{s}\left(X\right),\;G\;\left(\phi_{3\times 3}^{\mathrm{DW}}\phi_{3\times 3}^{\mathrm{DW}}\phi_{s}\left(X\right)\right)\right)\right)$$

where $\phi_{s}\left(\cdot \right)$ projects the input to the C/2-dimensional intermediate subspace, $\phi_{3\times 3}^{\mathrm{DW}}$ is a 3×3 depthwise separable convolution, G(·) is the GELU activation function, and Shuffle(·) is the channel shuffle operation.

### 3.3. Adaptive Multi-Scale Recalibrated Decoupled Detection Head

**Figure 4:**
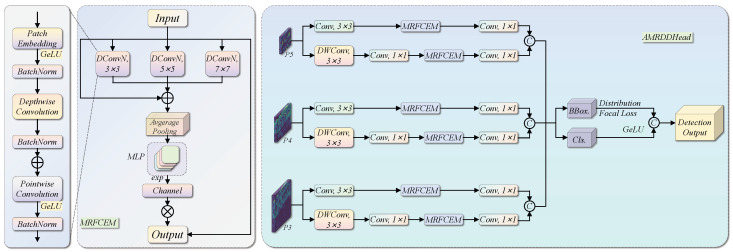
Structure of the AMRDDHead. The diagram illustrates the overall architecture and prediction pipeline of AMRDDHead, showing MRFCEM embedded in the decoupled bounding-box regression and category classification branches for channel-adaptive recalibration.

Classification and regression branches impose inherently different perceptual demands on feature channels: classification requires channels encoding category-discriminative texture and appearance, whereas regression requires channels encoding spatial boundary gradients and geometric structure. The standard YOLO detection head uses a shared, non-adaptive feature representation that cannot simultaneously satisfy both requirements, particularly under the high inter-class visual similarity characteristic of the MAR20 dataset. AMRDDHead addresses this by embedding MRFCEM into both branches to implement content-adaptive channel recalibration, dynamically suppressing background-redundant channels and reinforcing those encoding discriminative aircraft structure. The structure is illustrated in Figure 4.

AMRDDHead inherits the Detect_SEAM framework and replaces the original Squeeze-and-Excitation Attention Module (SEAM) with MRFCEM, preserving the decoupled detection head structure while enhancing multi-scale channel adaptability. Both branches share a common pipeline: projection to an intermediate feature space, MRFCEM channel recalibration, and a 1×1 convolution for final prediction. They differ in input processing and output dimensionality. The regression branch projects the *i*-th scale feature map $X_{i}\in R^{B\times C_{i}\times H_{i}\times W_{i}}$ to the $c_{2}$-dimensional space via a standard 3×3 convolution, applies MRFCEM, and outputs a $4\times r_{\max}$-dimensional distributed bounding-box encoding:

(11) $${\hat{b}}_{i}=\phi_{\mathrm{reg}}^{1\times 1}\;\left(M\;\left(\phi_{\mathrm{reg}}^{3\times 3}\left(X_{i}\right)\right)\right)\in R^{B\times 4r_{\max}\times H_{i}\times W_{i}}$$

The classification branch instead uses a lightweight combination of depthwise convolution (DWConv) and pointwise convolution for input compression, applies MRFCEM, and outputs a category confidence map:

(12) $${\hat{c}}_{i}=\phi_{\mathrm{cls}}^{1\times 1}\;\left(M\;\left(\phi_{\mathrm{cls}}^{\mathrm{PW}}\;\left(\phi_{\mathrm{DW}}^{3\times 3}\left(X_{i}\right)\right)\right)\right)\in R^{B\times N_{c}\times H_{i}\times W_{i}}$$

where M(·) denotes the MRFCEM channel attention operation, $r_{\max}=16$ is the DFL distribution range, and $N_{c}$ is the number of target categories.

After concatenating predictions from all three detection scales, the bounding-box encoding vectors are decoded by the DFL layer to obtain continuous bounding-box coordinates:

(13) $$d_{i}=\mathrm{dist}2\mathrm{bbox}\;\left(\mathrm{DFL}\left({\hat{b}}_{i}\right),\;A_{i},\;\mathrm{xywh}\right)\times s_{i}$$

where $A_{i}$ are the anchor coordinates at scale *i* and $s_{i}$ is the corresponding stride scaling factor. The final output of the classification branch is mapped to a probability space by a Sigmoid activation function, and concatenated with the decoded bounding-box coordinates to form the complete detection prediction tensor:

(14) $$Y=\mathrm{Cat}\;\left(d,\;\sigma (c)\right)\in R^{B\times \left(4+N_{c}\right)\times N_{\mathrm{anchor}}}$$

where σ(·) is the element-wise Sigmoid function and $N_{\mathrm{anchor}}=\sum_{i}H_{i}W_{i}$ is the total number of anchors across all scales.

MRFCEM extends the classic Squeeze-and-Excitation (SE) channel attention by introducing multi-scale depthwise convolutional feature aggregation. Three parallel depthwise convolutional branches extract local spatial features at different receptive fields using patch sizes of 3×3, 5×5, and 7×7, respectively. Their outputs, together with the original input features, are globally average-pooled and averaged to produce fused multi-scale channel statistics:

(15) $$z=\frac{1}{4}\;\left(\mathrm{GAP}\left(\phi_{0}\left(X\right)\right)+\mathrm{GAP}\left(\phi_{1}\left(X\right)\right)+\mathrm{GAP}\left(\phi_{2}\left(X\right)\right)+\mathrm{GAP}\left(X\right)\right)\in R^{B\times C}$$

where $\phi_{i}\left(\cdot \right)$ denotes the *i*-th depthwise convolutional branch (i∈{0,1,2}, corresponding to patch sizes of 3, 5, and 7, respectively) and GAP(·) denotes global average pooling.

The fused statistics *z* are passed through a two-layer fully connected gating network to generate channel recalibration weights. These weights are exponentially amplified and multiplied element-wise with the original feature map. The exponential function ensures that recalibration weights are strictly positive and centred around unity, so that the default behaviour preserves the input unchanged:

(16) $$M\left(X\right)=X⊙\exp\;\left(W_{2}\;\delta \;\left(W_{1}z\right)\;1_{C\times C}\right)$$

where $W_{1}\in R^{(C/r)\times C}$ and $W_{2}\in R^{C\times (C/r)}$ are the learnable weight matrices, r=16 is the reduction ratio, δ(·) is the ReLU activation function, and ⊙ denotes broadcasted element-wise multiplication.

The parameter increase introduced by AMRDDHead is concentrated in the two-layer fully connected gating network within MRFCEM. For a feature map with *C* channels and reduction ratio r=16, each MRFCEM instance contributes $2C^{2}/r$ parameters from $W_{1}$ and $W_{2}$. Since MRFCEM is deployed independently in both the regression and classification branches at each of the three detection scales, the total gating network parameter count is $2\times 3\times 2C^{2}/r$. This growth is entirely dedicated to branch-specific frequency-selective recalibration rather than generic feature transformation, preserving the lightweight structure of the spatial feature extraction path.

## 4. Experimental Results and Analysis

### 4.1. Dataset Description

(1) MAR20 [1] is currently the largest remote sensing dataset for military aircraft recognition. It contains 3842 images, 20 target categories, and 22,341 annotated instances spanning SU-35, C-130, C-17, C-5, F-16, TU-160, E-3, B-52, P-3C, B-1B, E-8, TU-22, F-15, KC-135, F-22, FA-18, TU-95, KC-10, SU-34, and SU-24. All images were collected from Google Earth across 60 military airfields worldwide. The official partition provides 1331 training images and 2511 test images, with each instance annotated by both horizontal and rotated bounding boxes. The dataset features high inter-class appearance similarity and significant intra-class variation due to weather, season, illumination, and occlusion. In our experiments, the training set is further split into training, validation, and test subsets in a 7:1:2 ratio (931/133/267 images) using stratified random sampling, with all images from the same airfield assigned to a single subset to prevent data leakage. The category distribution across subsets deviates by no more than ±2% from the overall dataset proportions for each category. To verify that the reported gains are robust across evaluation protocols, we additionally evaluate YOLO11n and FAMA-DET under the official train/test partition.

(2) CORS-ADD [11] was released by Shi et al. in 2023 as a benchmark for cross-domain generalisation evaluation. It contains 5486 images from WorldView-2, WorldView-3, Pleiades, Jilin-1, IKONOS, and Google Earth, with 32,285 rotated bounding-box annotations covering four coarse-grained categories: civil aircraft, bombers, fighters, and airborne early-warning aircraft. Target scale ranges from 4×4 to 240×240 pixels across ramp, runway, carrier deck, sea surface, and airborne scenarios. After training on MAR20 under the 20-class fine-grained recognition setting, we conduct zero-shot transfer testing on CORS-ADD to evaluate cross-domain generalisation.

### 4.2. Experimental Environment and Parameter Settings

All experiments use a unified hardware and software environment. The CPU is an AMD EPYC 7T83 (24 vCPUs) with 90 GB of memory, and the GPU is an NVIDIA vGPU with 32 GB VRAM. The operating system is Ubuntu 22.04 LTS; the deep learning framework is PyTorch 2.5.1 with Python 3.10.12, CUDA 12.4, and cuDNN 9.1.0; all detection models are managed and trained within the Ultralytics framework.

Key training hyperparameters are: batch size 32, input resolution 640×640, 400 training epochs, initial learning rate (lr0) 0.02, momentum 0.937, and weight decay 0.0005. The SGD optimiser is paired with cosine annealing learning rate scheduling (cos_lr) for stable convergence in the later training phase. The warmup phase spans three epochs with an initial warmup momentum of 0.8. Data augmentation includes mosaic augmentation, random scaling, random flipping, and HSV colour space jitter. All remaining hyperparameters follow the official YOLO11n default configuration to ensure fairness and reproducibility.

### 4.3. Evaluation Metrics

To comprehensively evaluate detection accuracy, computational efficiency, and inference speed, the following metrics are adopted. Precision quantifies the proportion of correctly identified positive instances among all predictions:

(17) $$P=\frac{\mathrm{TP}}{\mathrm{TP}+\mathrm{FP}}$$

Recall quantifies the proportion of correctly identified positive instances among all ground-truth positives:

(18) $$R=\frac{\mathrm{TP}}{\mathrm{TP}+\mathrm{FN}}$$

The F1 score is the harmonic mean of precision and recall, providing a balanced measure under class imbalance:

(19) $$F1=\frac{2\mathrm{PR}}{P+R}$$

where TP, FP, and FN denote true positives, false positives, and false negatives, respectively. To capture localisation quality across varying IoU criteria, mean average precision at IoU threshold 0.5 (mAP50) and mean average precision averaged over IoU thresholds from 0.5 to 0.95 in steps of 0.05 (mAP50-95) are further adopted. Computational cost is quantified by giga floating-point operations (GFLOPs) and total trainable parameter count (Params) to reflect deployment feasibility, and inference throughput is measured by frames per second (FPS) to assess real-time applicability.

### 4.4. Ablation Experiments

#### 4.4.1. Intra-Module Ablation of FDACFE

To validate each key component in FDACFE, we examined the synergistic effect of CSSMConv and DKSC, and the impact of replacing different backbone positions with FDACFE. As shown in Table 1, five variants are evaluated, ranging from CSSMConv alone to full FDACFE deployment across different layer combinations.

CSSMConv alone yields modest recall and mAP50 gains, confirming that partial-channel frequency-aware mixing provides incremental representational benefit. The addition of DKSC, which shifts kernel parameterisation entirely into the frequency domain and conditions weight aggregation on input content, produces the dominant accuracy improvement, identifying content-adaptive spectral parameterisation as the primary driver of FDACFE’s discriminative capacity. Restricting replacement to P3, P4, and P5 outperforms the full P2–P5 scheme by 0.8% in mAP50, as including P2 disrupts the fine-grained spatial detail preserved in low-level features that is essential for localising small targets, confirming that frequency-domain adaptation is most beneficial at semantically richer intermediate and high-level stages.

#### 4.4.2. Key-Component Ablation of SABFPN

To validate each key component in SABFPN, we conducted a stepwise internal ablation focused on the independent contributions and synergistic gains of ACSU and the subcomponents of TEMSConv. As shown in Table 2, four variants are evaluated, starting from ACSU alone and progressively adding the depthwise separable convolution branch and channel shuffle operation of TEMSConv.

ACSU’s scale-normalised amplitude compensation improves strict localisation quality, reflected by a mAP50-95 improvement of 0.4 percentage points, while the marginal precision trade-off is attributable to increased recall sensitivity that recovers previously suppressed true positives. The addition of TEMSConv’s depthwise branches substantially improves recall by 1.2 % over ACSU alone, demonstrating that broadening the downsampling receptive field is critical for capturing morphologically diverse aircraft structures. The channel shuffle operation provides further synergistic gain by cross-mixing features from the two depthwise branches, whose differential receptive fields are complementary: the shallower branch encodes fine edge geometry while the deeper branch captures global shape statistics, and mixing both prior to concatenation ensures that neither is discarded during downstream fusion.

#### 4.4.3. Overall Ablation Study

**Figure 5:**
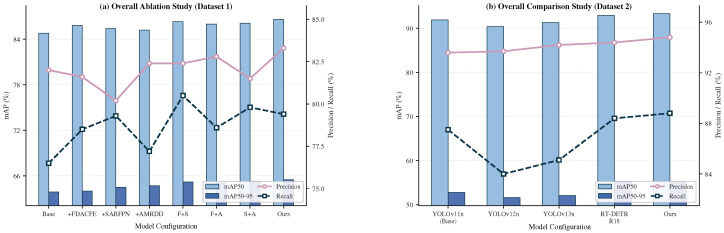
Performance visualisation: (**a**) Overall ablation study on the MAR20 dataset; (**b**) Cross-domain comparison with mainstream detectors on the CORS-ADD dataset.

We conducted a systematic ablation study to disentangle the standalone contributions of the three core modules from their combined synergistic effects. To verify that the gain of AMRDDHead originates from the multi-receptive-field channel recalibration mechanism rather than from increased parameter count, we include a parameter-controlled baseline that replaces MRFCEM with a standard Squeeze-and-Excitation (SE) module under the FDACFE + SABFPN configuration, with its reduction ratio adjusted such that the resulting model parameter count matches that of the full FAMA-DET. Results are presented in Table 3 and Figure 5a.

Among single-module configurations, FDACFE is the only component that simultaneously improves accuracy and reduces GFLOPs, a consequence of the partial-channel strategy in CSSMConv that confines frequency-domain computation to the most spectrally informative channels. SABFPN delivers accuracy gains at negligible computational overhead by replacing arithmetic operations in the upsampling step with a normalisation constant rather than adding convolutional layers. AMRDDHead achieves the highest single-module mAP50-95 improvement; at the same parameter count of 4.26 M as the full FAMA-DET, the parameter-controlled SE baseline FDACFE + SABFPN + SE achieves only 84.9% mAP50, which is 1.7% below the 86.6% mAP50 attained by the FDACFE + SABFPN + MRFCEM configuration. This confirms that the gain originates from multi-receptive-field statistics aggregation rather than from increased capacity. The consistent synergistic gains across all pairwise combinations reflect the complementary nature of the three modules: FDACFE enriches the spectral quality of backbone features, SABFPN preserves the amplitude and structural integrity of those features across scales, and AMRDDHead selectively recalibrates the resulting multi-scale representations for the distinct perceptual demands of regression and classification. Each module addresses a distinct architectural bottleneck, and their joint deployment in FAMA-DET achieves the best accuracy–efficiency trade-off among all configurations.

**Figure 6:**
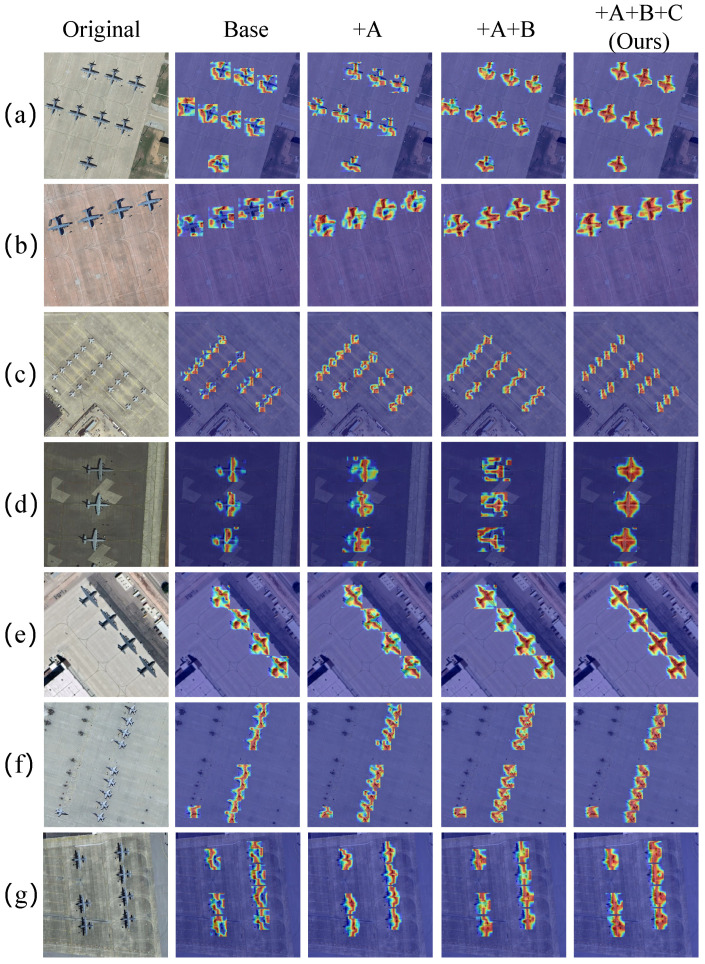
Attention heatmap visualisation on the MAR20 dataset. (**a**–**g**) represent seven typical optical remote sensing object detection scenarios. A denotes FDACFE, B denotes SABFPN, and C denotes AMRDDHead.

Attention heatmap analysis of progressive configurations (Baseline, +FDACFE, +FDACFE + SABFPN, and full FAMA-DET) is shown in Figure 6. Baseline activations are scattered across background regions; the progressive introduction of modules consistently concentrates responses onto the aircraft body while suppressing false activations, providing visual confirmation of the mechanism described analytically above. Two scenarios are particularly illustrative of the individual module contributions. In scenario (d), where wing-spread features dominate, FAMA-DET extends coverage from sparse fuselage activations to a complete cross-shaped high-response region spanning both fuselage and wings, demonstrating that FDACFE’s content-adaptive spectral parameterisation captures extended structural features rather than local texture patches; in scenario (f), where multiple aircraft are longitudinally aligned along the runway, FAMA-DET produces independent, clearly delineated high-activation regions for each individual aircraft, demonstrating that SABFPN’s amplitude normalisation prevents nearby high-level semantic features from bleeding across instance boundaries during fusion.

### 4.5. Comparison Experiments

#### 4.5.1. Backbone Feature Extraction Module Comparison

To validate the competitive advantage of FDACFE among backbone feature extraction modules, we conducted a lateral comparison with several existing C3k2 improvement strategies: C3k2_DCNv4 with deformable convolution [29], C3k2_RepConv with multi-branch reparameterisation [30], C3k2_DRG with lightweight design [31], and C3k2_RMBC with plain convolution [32]. To substantiate the claimed frequency-domain representational advantage, we additionally integrate two representative frequency-domain methods into the same C3k2 backbone slot under identical training settings: C3k2_FFC replaces the inner convolution with Fast Fourier Convolution [24], applying real-valued FFT to feature activations to achieve image-wide receptive fields; C3k2_GFNet substitutes the spatial mixing operation with learnable 2D FFT-domain filters following GFNet [25]. Both methods transform feature activations using fixed or globally shared frequency bases without adapting the frequency representation to individual input content, in contrast to DKSC’s content-adaptive kernel parameterisation in the frequency domain. Table 4 provides a comprehensive comparison under unified training and evaluation settings.

DCNv4 and RepConv improve mAP50 by up to 0.5% over the C3k2 baseline through deformable sampling and multi-branch reparameterisation, respectively, but incur 8–13% higher GFLOPs; DRG and RMBC reduce computation through lightweight redesign at the cost of up to 0.9% mAP50 regression. FFC and GFNet, which both operate on feature activations in the spectral domain, improve mAP50 by 0.2–0.4% over the C3k2 baseline, demonstrating that frequency-domain processing confers genuine representational benefits over purely spatial convolution; however, both apply frequency operations using fixed or globally shared bases, limiting their ability to adjust spectral emphasis per input sample. FDACFE surpasses these methods by additionally conditioning kernel weight aggregation on input content through the KSM, achieving the highest mAP50 of 85.8% while simultaneously reducing both GFLOPs and parameters relative to the C3k2 baseline, the only configuration to achieve this combination among all compared schemes.

#### 4.5.2. Feature Fusion Network Comparison

**Figure 7:**
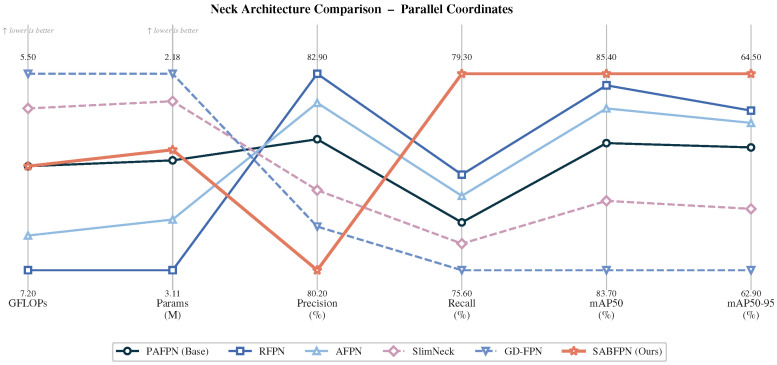
Parallel coordinate plot comparing detection performance of different feature fusion networks.

To validate the superiority of SABFPN among feature fusion networks, we systematically compared it with RFPN, AFPN, SlimNeck, and GD-FPN, with backbone and detection head fixed. Table 5 and Figure 7 present a comprehensive evaluation of detection accuracy, parameter count, and computational complexity.

RFPN and AFPN improve accuracy over PAFPN at the cost of 10–20% higher GFLOPs by introducing additional convolutional capacity in the fusion paths. SlimNeck and GD-FPN reduce computation but sacrifice up to 1.1% mAP50, indicating that indiscriminate capacity reduction in the neck degrades multi-scale representation quality for morphologically diverse aircraft. SABFPN is the only method that simultaneously improves both mAP50 and mAP50-95 without increasing GFLOPs or parameters, because its accuracy gain derives from correcting a systematic amplitude bias in the upsampling operator rather than from added convolutional capacity.

#### 4.5.3. Comparison with State-of-the-Art Methods

To evaluate FAMA-DET in the MAR20 aircraft detection task, we conducted a systematic comparison with mainstream methods from the DETR and YOLO series, assessing detection accuracy, computational complexity, and inference speed. To verify robustness across evaluation protocols, we additionally retrain YOLO11n and FAMA-DET strictly on the official MAR20 partition and report the results in the bottom region of Table 6.

The accuracy ceiling of DETR-series methods below 83.5% mAP50, despite exceeding 60 GFLOPs and requiring over 20 M parameters, reveals a fundamental mismatch between global attention-based token mixing and the spatially sparse, spectrally discriminative nature of aircraft features in remote sensing imagery; this computational overhead translates directly to sub-70 FPS throughput, precluding real-time deployment. Among YOLO-series methods, progressive architectural refinements from YOLOv8n to YOLOv13n narrow the accuracy gap, with YOLOv13n achieving 85.3% mAP50, the strongest prior work in this comparison. FAMA-DET surpasses YOLOv13n by 1.3% in mAP50 and 1.1% in mAP50-95 while reducing GFLOPs by 0.7, demonstrating that embedding frequency-domain adaptive mechanisms at each architectural level yields accuracy gains not obtainable by iterative spatial-domain refinement, even when controlling for computational cost.

Under the official partition, FAMA-DET improves YOLO11n by 1.7% mAP50 and 1.6% mAP50-95, consistent with the custom-split results.

**Figure 8:**
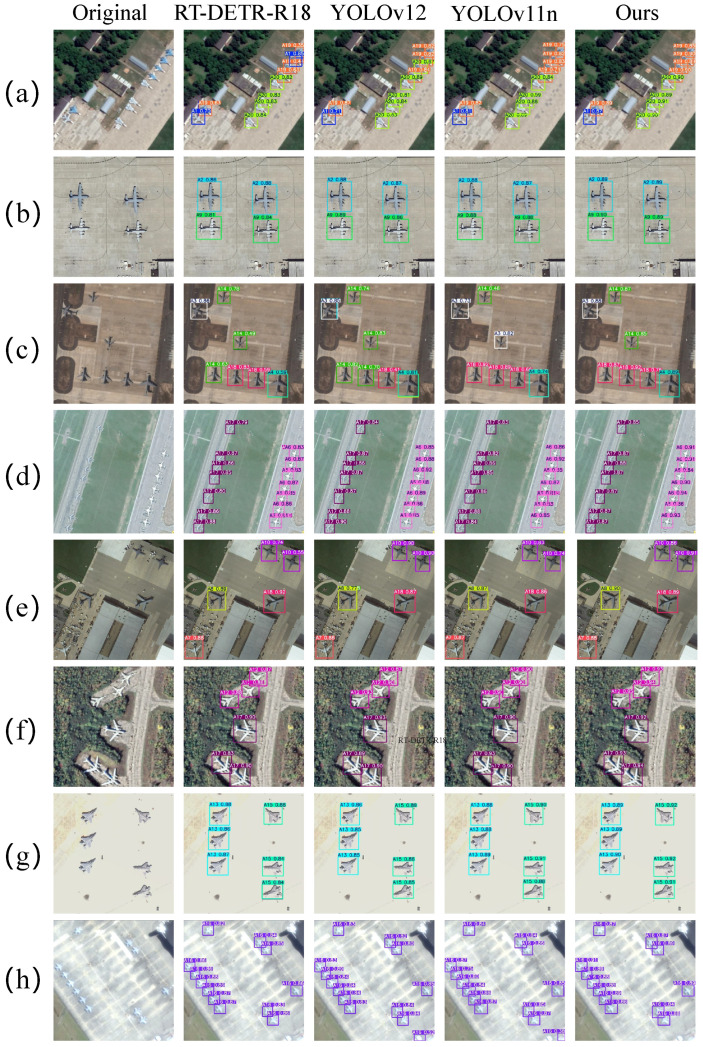
Visualisation comparison of detection results from different detectors on the MAR20 dataset. (**a**–**h**) represent eight typical optical target detection scenarios.

Figure 8 presents a visual comparison between FAMA-DET and three mainstream detectors across representative challenging conditions. The dominant pattern across scenarios is that spatial-domain methods exhibit degraded confidence and missed detections under density and scale variation, whereas FAMA-DET’s channel recalibration in AMRDDHead maintains stable discrimination regardless of instance count or arrangement. This advantage is most pronounced in small-target dense conditions. In scenario (g), where multiple small fighter jets are dispersed across the ramp, RT-DETR-R18 and YOLO11n produce missed detections, while FAMA-DET detects all instances with substantially higher confidence, attributable to SABFPN’s amplitude-normalised fusion preserving small-target boundary features that are otherwise attenuated during cross-scale propagation; in scenario (d), the same mechanism enables more precise bounding-box localisation under tight inter-instance spacing, where nearest-neighbour upsampling artefacts in competing methods cause adjacent instance boundaries to merge during feature fusion.

### 4.6. Cross-Domain Generalisation Experiments on CORS-ADD

#### 4.6.1. Ablation Study on the CORS-ADD Dataset

To validate the cross-domain generalisation effectiveness of the three core modules, we conducted zero-shot transfer ablation on CORS-ADD by progressively adding FDACFE, SABFPN, and AMRDDHead. Results are shown in Table 7.

FDACFE contributes the largest individual cross-domain gain, improving mAP50 by 0.7% and mAP50-95 by 0.9% with a simultaneous reduction in GFLOPs, identifying content-adaptive frequency-domain convolution as the primary source of cross-domain robustness. This result is consistent with the hypothesis that DKSC’s per-sample kernel adaptation learns sensor-invariant spectral patterns rather than sensor-specific spatial textures: by conditioning kernel weight aggregation on input content in Fourier space, FDACFE adjusts its spectral emphasis to the frequency characteristics of each input image, reducing sensitivity to the shift in sensor platform between MAR20 and CORS-ADD. SABFPN provides additional improvement by maintaining consistent amplitude relationships across feature scales regardless of source domain, and the full FAMA-DET achieves the best accuracy–efficiency balance among all configurations.

**Figure 9:**
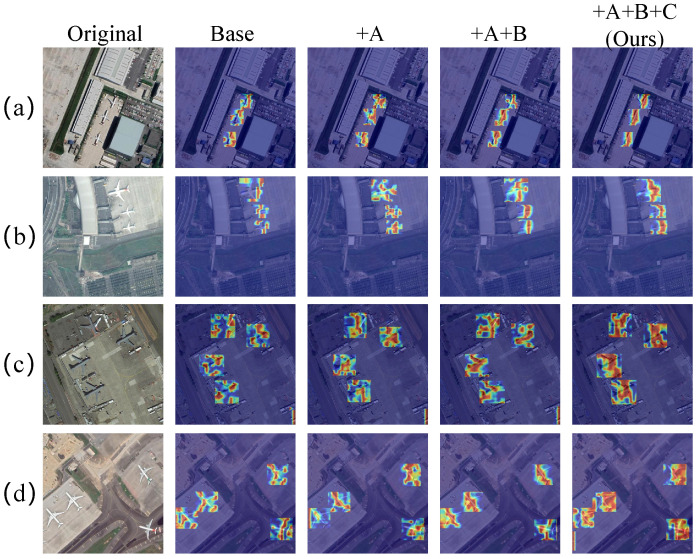
Attention heatmap visualisation on the CORS-ADD dataset. (**a**–**d**) represent four typical optical target detection scenarios. A denotes FDACFE, B denotes SABFPN, and C denotes AMRDDHead.

Attention heatmap analysis after progressively introducing each module is shown in Figure 9. The baseline generates extensive false activations on building rooftops and man-made structures that share local texture patterns with aircraft fuselages; this domain-induced confusion is systematically reduced as each module is added. Two scenarios illustrate the progression most clearly. In scenario (a), where aircraft and large buildings are spatially adjacent, the baseline’s inability to distinguish spectrally similar textures produces activation maps dominated by rooftop false positives, while FAMA-DET concentrates responses on the aircraft fuselage, demonstrating that frequency-domain adaptive convolution learns spectral discriminants that transfer across the rooftop–fuselage ambiguity without target-domain supervision; in scenario (c), with multiple aircraft at the apron under complex background structure, FAMA-DET produces clear and complete activation contours for each aircraft, confirming that SABFPN’s amplitude normalisation preserves instance-level boundary information under the denser background clutter of CORS-ADD.

#### 4.6.2. Cross-Domain Performance Comparison with Mainstream Detection Algorithms

Table 8 and Figure 5b compare FAMA-DET with YOLOv12n, YOLOv13n, and RT-DETR-R18 under unified zero-shot transfer evaluation on CORS-ADD.

YOLOv12n and YOLOv13n both experience mAP50 regression relative to the YOLO11n baseline under domain shift, declining by 1.5 and 0.6 %, respectively, indicating that their spatial-domain architectural refinements, which improve in-domain accuracy, do not generalise to unseen sensor characteristics. RT-DETR-R18 recovers cross-domain accuracy with a mAP50 improvement of 1.0 % over the baseline, suggesting that global attention-based representations capture some domain-invariant structure, but at the cost of 56.9 GFLOPs and 19.87M parameters. FAMA-DET achieves the highest mAP50 of 93.3% and the highest recall of 88.8% among all compared methods at only 5.4 GFLOPs, a 91.0% reduction in computation relative to RT-DETR-R18, demonstrating that per-sample frequency-domain adaptation provides a more parameter-efficient pathway to cross-domain robustness than global attention.

**Figure 10:**
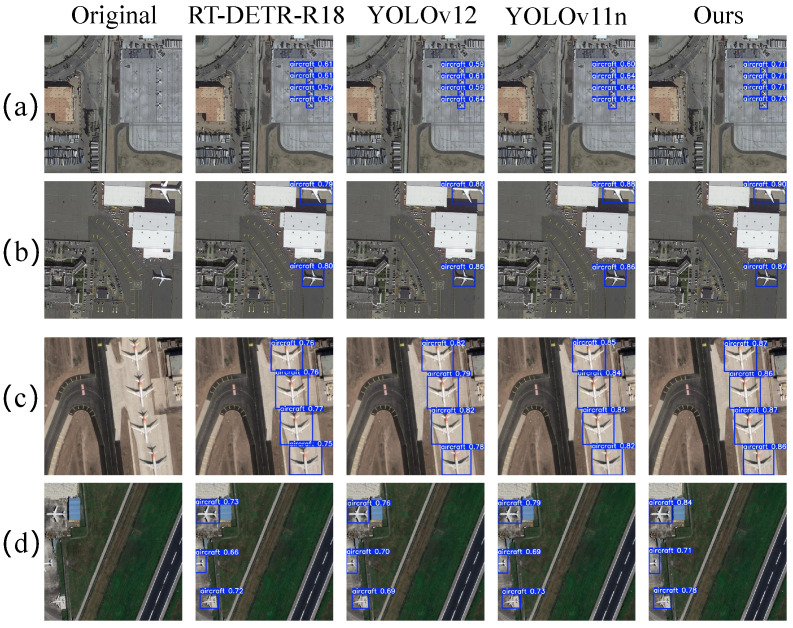
Visualisation comparison of cross-domain detection results from different detectors on the CORS-ADD dataset. (**a**–**d**) represent four typical optical target detection scenarios.

Figure 10 presents a visual comparison across four scenario types that are particularly challenging under domain shift: ramp parking near buildings, runway taxiing, building occlusion, and distant small targets. The dominant failure mode of comparison methods is confidence degradation under background interference from man-made structures with aircraft-like local textures, a challenge amplified by the sensor diversity of CORS-ADD. FAMA-DET consistently assigns higher and more stable confidence across all scenario types, with the most pronounced advantage in building-proximate and small-target conditions. In scenario (a), FAMA-DET improves detection confidence by 0.10–0.14 relative to RT-DETR-R18, and in scenario (d), FAMA-DET improves confidence by up to 0.18 over comparison methods, quantitatively confirming that frequency-domain adaptive design reduces confusion between structural background textures and aircraft spectral signatures.

## 5. Conclusions

FAMA-DET embeds content-adaptive spectral representation across the backbone, neck, and detection head through three coordinated modules. On MAR20, the model achieves superior accuracy–efficiency trade-off over all compared methods at 5.4 GFLOPs with real-time throughput; under zero-shot transfer to CORS-ADD, it attains the highest mAP50 at 91.0% fewer GFLOPs than RT-DETR-R18. Results are consistent across both the custom stratified and official MAR20 partitions.

Limitations and future directions are threefold. Horizontal bounding-box supervision in the current framework does not exploit the rotated annotations available in MAR20, leaving oriented detection as an immediate extension. The parameter overhead introduced by MRFCEM constrains deployment on severely resource-limited platforms, motivating further compression. Finally, generalisation to ship and vehicle categories remains unvalidated, and cross-task evaluation is planned. 

## Figures and Tables

**Table 1:** Ablation results of internal modules in FDACFE. P2–P5 denote the four backbone output stages at strides of 4, 8, 16, and 32 pixels, respectively. ✓ and – indicate the inclusion and exclusion of the corresponding module, respectively.

CSSMConv	DKSC	Backbone Position	P (%)	R (%)	F1 (%)	mAP50 (%)	mAP50-95 (%)
–	–	–	82.0	76.5	79.2	84.8	63.9
✓	–	P3, P4, P5	82.3	77.1	79.6	85.1	64.2
✓	✓	P2, P3, P4, P5	82.1	76.9	79.4	85.0	64.1
✓	✓	P2, P3, P4	82.5	77.5	79.9	85.3	63.8
✓	✓	P3, P4, P5	81.6	78.5	80.0	85.8	64.0

**Table 2:** Ablation results of key components in SABFPN. ✓ and – indicate the inclusion and exclusion of the corresponding component, respectively.

ACSU	TEMSConv-DWConv	TEMSConv-Shuffle	P (%)	R (%)	mAP50 (%)	mAP50-95 (%)
–	–	–	82.0	76.5	84.8	63.9
✓	–	–	81.6	77.4	84.9	64.3
✓	✓	–	81.2	78.6	85.2	64.1
✓	✓	✓	80.2	79.3	85.4	64.5

**Table 3:** Overall ablation results. ✓ and – indicate the inclusion and exclusion of the corresponding module or component, respectively.

FDACFE	SABFPN	AMRDDHead		GFLOPs	Params (M)	P (%)	R (%)	mAP50 (%)	mAP50-95 (%)
		MRFCEM	SE						
–	–	–	–	6.3	2.59	82.0	76.5	84.8	63.9
✓	–	–	–	5.7	2.29	81.6	78.5	85.8	64.0
–	✓	–	–	6.3	2.54	80.2	79.3	85.4	64.5
–	–	✓	–	6.1	5.23	82.4	77.2	85.2	64.7
✓	✓	–	–	5.7	2.25	82.4	80.5	86.3	65.2
✓	–	✓	–	5.5	4.81	82.8	78.6	86.0	65.1
–	✓	✓	–	6.0	5.18	81.5	79.8	86.1	65.3
✓	✓	–	✓	5.4	4.26	82.0	76.8	84.9	64.2
✓	✓	✓	–	5.4	4.26	83.3	79.4	86.6	65.5

**Table 4:** Comparison of different C3k2 improvement strategies.

Model	GFLOPs	Params (M)	P (%)	R (%)	mAP50 (%)	mAP50-95 (%)
C3k2 (base)	6.3	2.59	82.0	76.5	84.8	63.9
C3k2_DCNv4 [29]	7.1	2.98	82.8	77.1	85.3	64.1
C3k2_RepConv [30]	6.8	2.76	82.4	76.8	85.1	63.7
C3k2_DRG [31]	5.9	2.21	81.2	75.9	84.3	63.2
C3k2_RMBC [32]	5.5	2.08	80.7	75.3	83.9	62.8
C3k2_FFC [24]	6.9	2.74	81.9	77.2	85.2	64.0
C3k2_GFNet [25]	6.7	2.68	81.6	76.9	85.0	63.8
FDACFE (Ours)	5.7	2.29	81.6	78.5	85.8	64.0

**Table 5:** Comparison of different feature fusion networks.

Model	GFLOPs	Params (M)	P (%)	R (%)	mAP50 (%)	mAP50-95 (%)
PAFPN (base)	6.3	2.59	82.0	76.5	84.8	63.9
RFPN [33]	7.2	3.11	82.9	77.4	85.3	64.2
AFPN [34]	6.9	2.87	82.5	77.0	85.1	64.1
SlimNeck [35]	5.8	2.31	81.3	76.1	84.3	63.4
GD-FPN [36]	5.5	2.18	80.8	75.6	83.7	62.9
SABFPN (Ours)	6.3	2.54	80.2	79.3	85.4	64.5

**Table 6:** Comprehensive performance comparison with mainstream state-of-the-art models.

Model	GFLOPs	Params (M)	P (%)	R (%)	mAP50 (%)	mAP50-95 (%)	FPS
DETR-Based Object Detectors							
RT-DETR-R18 [10]	60.2	20.03	81.3	74.6	82.8	61.2	69.2
RT-DETR-R34 [10]	92.1	31.08	82.1	75.3	83.5	62.0	58.6
DN-DETR [37]	94.5	40.35	80.6	73.8	82.1	60.7	31.3
DAB-DETR [38]	101.2	43.70	79.8	72.9	81.4	59.8	29.7
DINO-DETR [39]	107.4	47.54	80.2	73.5	82.6	61.0	27.8
One-Stage Object Detectors							
YOLOv5n [40]	4.5	1.87	78.4	71.2	80.3	58.1	118.3
YOLOv6-n [41]	11.4	4.63	80.5	74.2	83.1	60.8	105.6
YOLOv7-t [42]	13.2	6.01	80.9	74.7	83.4	61.2	98.4
YOLOv8n [43]	8.7	3.16	81.1	75.0	83.7	61.4	111.2
YOLOv9t [44]	7.7	2.01	81.8	75.6	84.1	62.0	108.5
YOLOv10n [45]	6.7	2.73	81.5	75.3	83.9	61.7	113.1
Gold-YOLO [46]	12.1	5.62	82.3	76.1	84.5	62.8	94.3
YOLO-MS-XS [47]	8.2	3.08	81.7	75.8	84.2	62.3	102.7
YOLOv12n [48]	6.5	2.64	82.2	76.8	85.0	64.2	113.8
YOLOv13n [49]	6.1	2.48	82.4	77.1	85.3	64.4	116.1
YOLO11n (base) [26]	6.3	2.59	82.0	76.5	84.8	63.9	115.4
FAMA-DET (Ours)	**5.4**	4.26	**83.3**	**79.4**	**86.6**	**65.5**	109.7
Official Partition Protocol							
YOLO11n [26]	6.3	2.59	81.3	75.6	83.9	63.1	115.4
FAMA-DET (Ours)	**5.4**	4.26	**82.6**	**78.5**	**85.6**	**64.7**	109.7

Bold indicates the best performance.

**Table 7:** Ablation results of the proposed core modules on the CORS-ADD dataset. The symbols “✓” and “–” indicate the inclusion and exclusion of the corresponding module, respectively.

FDACFE	SABFPN	AMRDDHead	GFLOPs	Params (M)	P (%)	R (%)	mAP50 (%)	mAP50-95 (%)
–	–	–	6.3	2.59	93.6	87.5	91.9	52.8
✓	–	–	5.7	2.29	94.1	88.5	92.6	53.7
✓	✓	–	5.7	2.25	94.4	88.4	92.9	54.5
✓	✓	✓	5.4	4.26	94.8	88.8	93.3	55.1

**Table 8:** Cross-domain generalisation performance comparison on the CORS-ADD dataset.

Model	GFLOPs	Params (M)	P (%)	R (%)	mAP50 (%)	mAP50-95 (%)
YOLO11 (base) [26]	6.3	2.59	93.6	87.5	91.9	52.8
YOLOv12n [48]	6.5	2.64	93.7	84.0	90.4	51.6
YOLOv13n [49]	6.1	2.48	94.2	85.1	91.3	52.1
RT-DETR-R18 [10]	60.2	20.03	94.4	88.4	92.9	55.3
FAMA-DET (Ours)	**5.4**	4.26	**94.8**	**88.8**	**93.3**	55.1

Bold indicates the best performance.

## Data Availability

The MAR20 dataset is a publicly available dataset introduced in [1]; however, the original link is currently inaccessible. Interested researchers can contact the authors of the original publication for access. The CORS-ADD dataset is publicly available at https://github.com/sgtojd/CORS-ADD-Complex-Optical-Remote-Sensing-Aircraft-Detection-Dataset (accessed on 20 December 2025).
